# Automating evaluation of LLM-generated responses to patient questions about rare diseases

**DOI:** 10.1093/jamiaopen/ooag054

**Published:** 2026-04-17

**Authors:** Min Zhao, Inez Y Oh, Aditi Gupta, Sally Cohen-Cutler, Kathryn M Harmoney, Albert M Lai, Bryan A Sisk

**Affiliations:** Institute for Informatics, Data Science and Biostatistics, Washington University in St. Louis School of Medicine, St. Louis, MO, 63110, United States; Institute for Informatics, Data Science and Biostatistics, Washington University in St. Louis School of Medicine, St. Louis, MO, 63110, United States; Institute for Informatics, Data Science and Biostatistics, Washington University in St. Louis School of Medicine, St. Louis, MO, 63110, United States; Pediatric Oncology, Children’s Hospital of Philadelphia, Philadelphia, PA, 19104, United States; Pediatric Oncology and Hematology, University of New Mexico’s Health Sciences Center, Albuquerque, NM, 87106, United States; Institute for Informatics, Data Science and Biostatistics, Washington University in St. Louis School of Medicine, St. Louis, MO, 63110, United States; Pediatric Hematology and Oncology, Washington University in St. Louis School of Medicine, 660 South Euclid Ave, St. Louis, MO, 63110, United States

**Keywords:** Large language models, automated evaluation, natural language processing, rare diseases, vascular anomaly

## Abstract

**Objectives:**

Patients with rare diseases often struggle to find accurate medical information, and large language model (LLM)-based chatbots may help meet this need. However, evaluating LLM-generated free-text answers typically requires physician review, which is time-consuming and difficult to scale. This study compared traditional natural language processing (NLP) metrics to emerging LLM-based evaluation approaches for assessing answer quality in the context of Complex Lymphatic Anomalies (CLAs).

**Materials and Methods:**

We compiled 25 common patients’ questions about CLAs and generated 175 responses to these questions from seven LLMs. Three expert physicians scored these responses for accuracy. We compared these physician-assigned scores with automated scores, generated by four NLP sentence similarity metrics (BLEU, ROUGE, METEOR, BERTScore) and six LLM evaluators (GPT-4, GPT-4o, Qwen3-32B, DeepSeek-R1-14B, Gemma3-27B, LLaMA3.3-70B). We examined both LLM-based scoring with and without reference answers (reference-guided vs reference-free). We calculated Spearman, Phi, and Kendall’s Tau correlation coefficients to assess alignment between automated and physician-assigned scores.

**Results:**

LLM-based evaluation demonstrated stronger alignment with physician-assigned scores than NLP metrics. The reference-guided GPT-4 evaluator achieved the highest correlation with physician-assigned scores (*ρ* = 0.758), followed by GPT-4o (*ρ* = 0.727). NLP metrics showed weak to moderate correlations with physician-assigned scores (*ρ* = 0.240–0.403). Reference-guided scoring outperformed reference-free methods.

**Discussion:**

Reference-guided LLM-based evaluation methods approximate expert physicians’ judgment better than traditional NLP metrics, offering an effective, scalable approach for assessing LLM-generated responses to patient questions about rare disease.

**Conclusion:**

LLM-based evaluation, particularly reference-guided scoring with GPT models, can support the scalable development and evaluation of LLM-based rare disease-specific chatbot systems.

## Background and significance

Rare diseases, as defined by the U.S. National Institutes of Health (NIH), are conditions that affect fewer than 200,000 people in the United States.^1^ Although individually rare, these diseases collectively affect millions of individuals worldwide and pose a significant burden on patients and their families.^2–4^ Rare disease patients experience barriers to accessing expert physicians to manage their care, and often struggle to find accurate and reliable information about their condition.^5–11^ In this study, as a model system for understanding information needs and communication barriers in rare diseases, we focused on Complex Lymphatic Anomalies (CLAs), a group of four rare diseases characterized by abnormal lymphatic development that can cause high morbidity and lifelong complications.^12^ Prior studies have shown that accessing high-quality information is essential for patients and families affected by CLAs to receive optimal care.^5^^,^^6^^,^^13^ In fact, patients who report receiving better information about their disease also report better mental health, physical health, and ability to navigate the healthcare system.^14^^,^^15^ Given the dearth of expert clinicians and lack of high-quality information, there is a critical need to develop accurate chatbots that can support the information needs of patients and families affected by CLAs.

Recent advancements in large language models (LLMs) offer a promising opportunity to develop scalable, patient-centered AI chatbot systems tailored to rare diseases like CLAs.^16^^,^^17^ LLMs are sophisticated AI algorithms trained on vast amounts of web-sourced data that can understand and generate human-like text.^18^ These LLM-based chatbots have the potential to expand access to trustworthy health information and support patient-engaged decision-making.^19^

However, LLMs may also produce inaccurate information, potentially leading to confusion, frustration, or inappropriate medical decisions.^16^^,^^20^ Accordingly, there is increasing emphasis on developing robust approaches for evaluating the quality of LLM-generated outputs.^19^^,^^21^

LLM-based chatbots usually generate responses in free-text form.^18^ Unlike structured or exam-style outputs constrained by predefined formats (eg, multiple-choice or checkbox options) that can be assessed against fixed gold-standard answers, free-text responses provide nuanced, contextualized, and personalized information, and thus require more sophisticated and flexible metrics to evaluate their quality. Although human evaluation remains the gold-standard for assessing answer quality and alignment with clinical expertise, it is time-consuming, resource-intensive, and difficult to scale consistently.^22^^,^^23^ Given LLM-based chatbots’ capacity to rapidly generate large volumes of text, scalable and effective methods to evaluate these responses are urgently needed. Therefore, investigating the efficacy of automated methods for evaluating the quality of LLM-generated free-text responses is critical.

Most efforts to automatically evaluate free-text answers have relied on traditional natural language processing(NLP) metrics.^22^ These include n-gram-based measures such as ROUGE, BLEU, and METEOR, as well as embedding-based semantic similarity metrics like BERTScore.^24–27^ Originally developed for tasks such as text summarization and machine translation, these metrics assess the degree of lexical or semantic similarity between a generated response and a reference answer.^24–26^ However, their effectiveness in evaluating nuanced answers to questions from rare disease patients remains uncertain.

An emerging alternative is LLM-based evaluation, also referred to as ***LLM-as-a-judge,*** which leverages a stronger or more reliable LLM, such as GPT-4, as a surrogate for humans to evaluate outputs generated by other models.^28^^,^^29^ This approach offers the potential for consistent and scalable evaluation and has shown promising results in evaluating general NLP tasks, such as long-form summarization and open-domain QA.^21^^,^^30–34^ However, their effectiveness for evaluating free-text responses to medical questions about rare diseases has not yet been investigated. To address this gap, we systematically assessed both traditional NLP metrics and LLM-based methods for evaluating LLM responses to patient questions about CLAs.

## Materials and methods

### CLA-QA dataset

We compiled a dataset consisting of 175 free-text responses generated by seven LLMs in response to 25 questions related to CLAs.

Question selection: A physician with expertise in CLAs (BAS) curated 25 questions about CLAs based on prior qualitative studies with patients and caregivers in this population, as well as “frequently asked questions” listed on the Lymphangiomatosis & Gorham’s Disease website, an advocacy group for patients with CLAs.^6^^,^^7^^,^^15^ The question set included four subtypes of CLAs: Gorham-Stout disease, Generalized Lymphatic Anomaly, Kaposiform Lymphangiomatosis, Central Conducting Lymphatic Anomaly, and covered topics on disease definition, diagnosis, treatment, cause, family support, and mental health (see Supplementary Table S1, available as supplementary data at [*JAMIA Open*] online).

Answer-generation models: To generate responses for evaluation, we queried a leading proprietary model and a diverse set of open-source LLMs. The proprietary model, GPT-4, has demonstrated strong medical reasoning capabilities and high accuracy in addressing patient-facing rare disease questions.^35–37^ The open-source models included LLaMA3.2-3.2B,^38^ DeepSeek-R1-8B,^39^ Phi-4-14B,^40^ Mistral-7B,^41^ Gemma 2-7B,^42^ and Qwen 2-7B,^43^ representing a range of model architectures and capabilities. Evaluating both proprietary and open-source models allowed for a broader assessment of model capabilities, including the potential of locally deployable LLMs for use in settings with cost, privacy, or security constraints.

Human annotation: Three domain-expert physicians specializing in CLAs (BAS, SCC, and KH) independently evaluated the accuracy of all 175 responses generated by the seven models. These clinicians were all board-certified pediatric hematologists and oncologists with expertise in vascular anomalies and complex lymphatic anomalies. All three clinicians serve in leadership at multidisciplinary vascular anomaly centers and have published studies in this population. Annotators were blinded to model identities, and the seven responses for each question were presented in randomized order, although the question sequence was fixed. **Accuracy** was chosen as the primary evaluation criterion for evaluating answer quality, given its critical importance in medical chatbots. Accuracy was defined as the factual correctness of a response based on clinical expertise, including whether the answer was medically accurate, free of misinformation, consistent with current standards of care, and supported by scientific or clinical evidence.^44^ Ratings were assigned using a 5-point Likert scale, where 1 indicated a completely inaccurate answer and 5 indicated a completely accurate answer. The mean accuracy score from the three physicians was used as the physician-assigned accuracy score for all analyses.

Reference answer selection: Many automatic evaluation methods, including traditional NLP sentence similarity metrics and LLM-based reference-guided scoring, require high-quality reference answers to enable consistent and scalable scoring of model outputs (described in detail later in MATERIALS AND METHODS-Automatic evaluation methods). Because expert-generated ideal answers are time-consuming to produce at scale, we instead identified the best-performing answer-generation model through expert evaluation and used its responses as reference answers for the reference-guided evaluation approaches. After selecting this model’s responses as the reference, we excluded them from subsequent automated evaluations.

### Automatic evaluation methods

#### NLP sentence similarity metrics

We used traditional NLP metrics to measure the similarity between generated responses and corresponding reference answers. Specifically, we evaluated responses using four commonly used metrics: ROUGE-L, BLEU, METEOR, and BERTScore.^24–27^ Detailed descriptions of theses metrics are provided in Supplementary Section S1, available as supplementary data at [*JAMIA Open*] online.These metrics served as proxies for answer quality by quantifying lexical or semantic similarity. Scores range from 0 and 1, with higher values reflecting greater similarity between the generated and reference answers.

#### LLM-based evaluation

We implemented two LLM-based evaluation methods to assess the quality of answers generated by different language models: reference-guided and reference-free scoring.

*Reference-guided scoring:* ^45^ ^,^ ^46^ The LLM evaluator was provided with a reference answer, along with the corresponding question and a model-generated response. The evaluator was then prompted to assign an accuracy score using a 5-point Likert scale, where higher scores indicate greater factual correctness. We prompted the LLMs to assess accuracy that aligned with the criterion used by physician annotators during human evaluation.

*Reference-free scoring:* ^47^ The LLM evaluator was only provided with the question and a model-generated response, without a reference answer. The evaluator was then asked to assign an accuracy score using the same 5-point Likert scale. To ensure consistency across evaluation conditions, we applied the same accuracy scoring criterion used in the human annotation and reference-guided evaluation.

**Figure 1.**
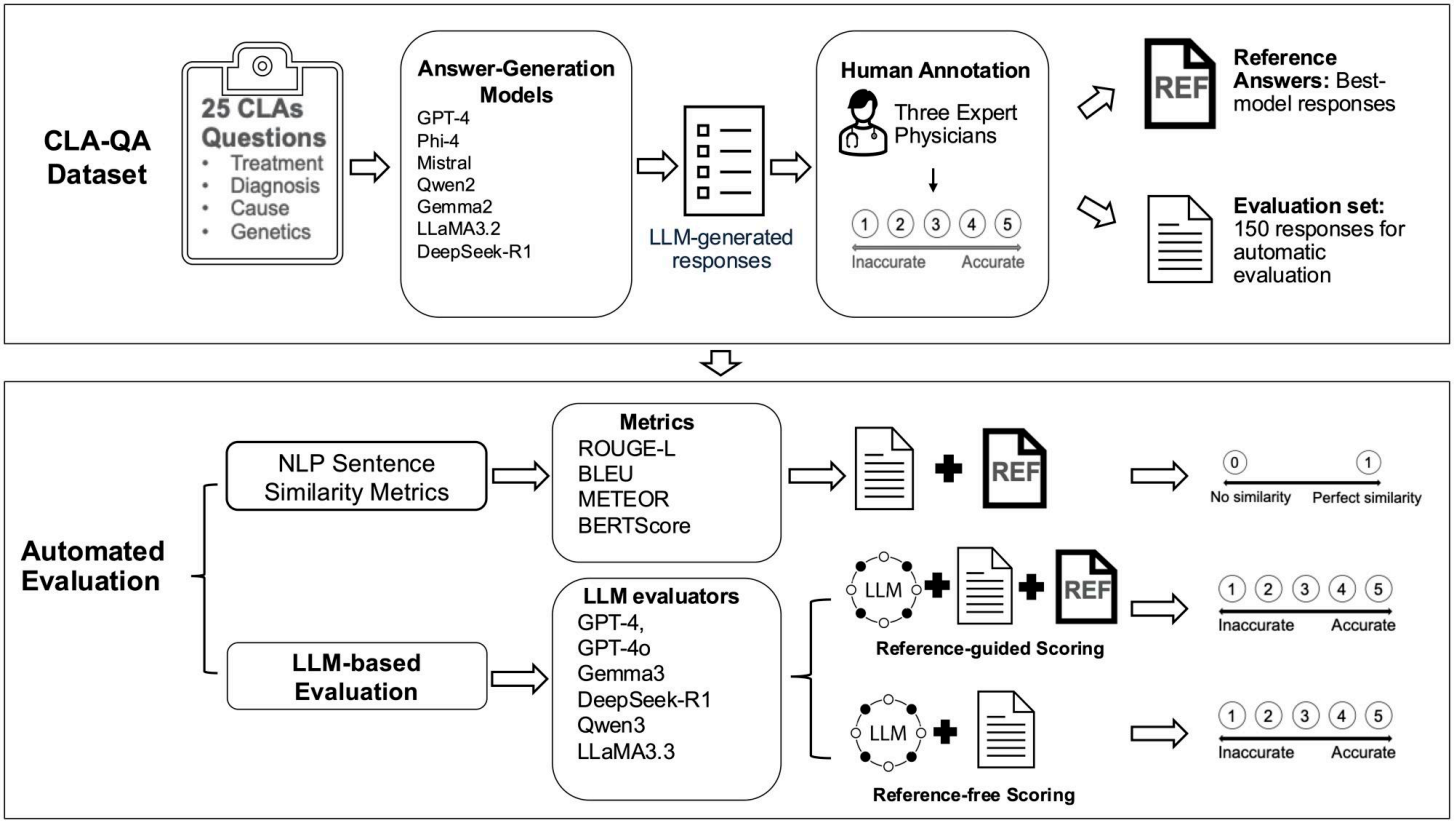
Overview of the workflow for automating evaluation of LLM-generated free-text responses.

We evaluated six LLM evaluators: GPT-4o,^48^ GPT-4,^36^ Gemma 3-27B,^49^ DeepSeek-R1-14B,^39^ Qwen 3-32B^50^ and LLaMA3.3-70B.^38^ Additional model information, including release date, parameter size, architecture, and availability (open-weight or proprietary), is provided in Supplementary Table S8. Figure 1 provides an overview of the workflow for automating this evaluation of LLM-generated free-text responses. Open-source LLMs were deployed locally using Ollama on Washington University’s Research Infrastructure Services computing platform. Ollama enables efficient local execution of open-weight LLMs.^51^ These models were run with 32 GB RAM and a single NVIDIA A100 GPU. The proprietary LLM evaluators included GPT-4 (version 2024-11-20) and GPT-4o (version 2024-08-06), accessed through Microsoft Azure’s OpenAI Service using a HIPAA-compliant subscription within Washington University’s Azure tenant. The same prompts were used for every LLM evaluator. LLM evaluators’ temperatures were set to 0.

**Figure 2.**
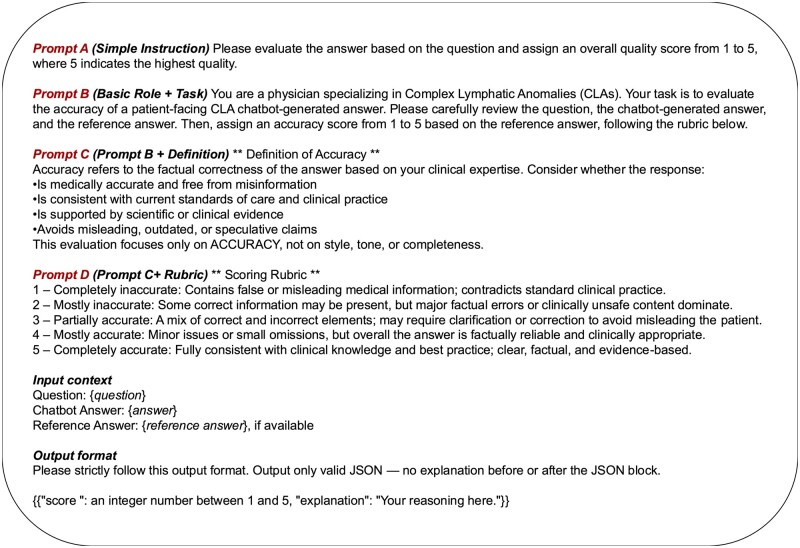
LLM-based Evaluation Prompt Design Four prompt variants were used to assess the sensitivity of LLM evaluators to prompt structure. Prompt A (Simple Instruction) requested an accuracy score from 1 to 5 without additional context. Prompt B (Role Specification) framed the LLM as a CLA physician and requested an accuracy score. Prompt C (Definition Prompt; default for subsequent analyses) provided a definition of accuracy and evaluation criteria. Prompt D (Definition + Rubric) further included a detailed 1–5 scoring rubric to promote more consistent interpretation.

*Evaluation design and robustness analysis in LLM-based evaluation:* To assess the stability of LLM-based evaluation, we conducted 50 iterations of reference-guided scoring using LLM evaluators. For every response, scores were generated 50 times per evaluator. The variance across iterations was calculated for each response, and then the mean variance and standard deviation were computed for each LLM evaluator. To examine the impact of prompt design, we evaluated four prompt (A–D) that differed in the level of structure and guidance provided to the LLM evaluator (Figure 2). Prompt A instructed the model to assign an accuracy score without additional context. Prompt B added a simple role specification. Prompt C defined accuracy and the evaluation criteria and served as the *default* prompt for subsequent analyses. Prompt D further included a detailed scoring rubric. We compared their performance by calculating the correlation between their assigned accuracy scores and physician-assigned scores. This analysis provided insight into the sensitivity of LLM evaluators to prompt structure and complexity.

We analyzed two common sources of bias in LLM-based evaluation: verbosity bias and self-enhancement bias. Verbosity bias, also known as length bias, refers to the tendency of LLM evaluators to favor responses of a particular length, often showing a preference for more verbose outputs.^52^ We tested this by computing the Spearman correlation between answer length and accuracy scores assigned by physicians and LLM evaluators under the reference-guided setting. Self-enhancement bias refers to the tendency of LLM evaluators to favor responses generated by themselves or by models similar to themselves.^21^ We assessed self-enhancement bias by comparing how each LLM evaluator scored responses generated by its corresponding model family.

### Statistical analysis

To assess inter-rater reliability among the three physicians, we computed the intraclass correlation coefficient (ICC) using a two-way random effects model for absolute agreement, ICC(2,1), which considering each rater as a random sample from a larger population of possible raters and evaluates the degree of absolute agreement among raters.^53^^,^^54^ To evaluate response-level associations, we used Spearman’s rank correlation coefficient (*ρ*) to assess the relationship between physician-assigned scores, traditional NLP metric scores, and LLM-assigned scores.^55^ We interpreted correlation strength using the following thresholds: 0.00–0.10 negligible, 0.10–0.39 weak, 0.40–0.69 moderate, 0.70–0.89 strong, and 0.90–1.00 very strong correlations.^56^ At the answer-generation model level, we calculated Kendall’s *τ* to assess correlations between the average physician-assigned scores and LLM-assigned accuracy scores. Kendall’s *τ* was selected because it is well suited for small sample sizes and rank-based comparisons.^57^ This analysis included six answer-generation models (*n = *6), each represented by its average physician-assigned score and corresponding average LLM evaluator score. We used descriptive statistics to summarize average NLP metric values, LLM-assigned accuracy scores, and inference time for each evaluator. To test differences in model scores across three or more related groups, we applied the Friedman test, a non-parametric test for detecting differences among multiple related groups, using a significance threshold of *p* < .05.^58^

To further evaluate the binary discrimination ability of LLM-based scoring methods, we recoded physician-assigned and LLM-assigned scores into binary labels, with scores of 1–3 categorized as low accuracy and 4–5 as high accuracy. We then calculated Phi correlation coefficients (*φ*) between the binary labels derived from physician-assigned scores and those derived from LLM-assigned scores, thereby assessing each LLM evaluator’s ability to distinguish between low- and high-quality responses.^59^^,^^60^ See detailed description of these statistical tests in Supplementary Section S2, available as supplementary data at [*JAMIA Open*] online. All statistical analyses were conducted using Python 3.12.

## Results

### Human annotation results

Inter-rater reliability among three expert physicians was high (ICC = 0.852), indicating substantial agreement across raters. An illustrative example of rater disagreement is provided in Supplementary Table S2, available as supplementary data at [*JAMIA Open*] online. In all subsequent analyses, “**physician-assigned score**” refers to the mean accuracy score from three expert physicians per response.

**Figure 3.**
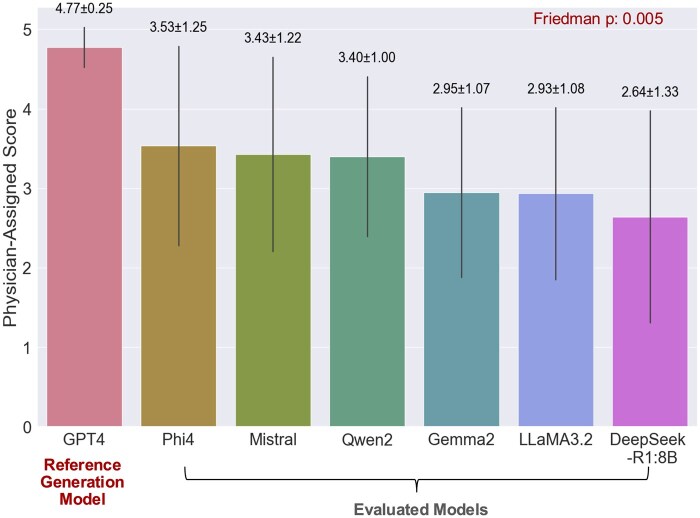
Comparison of LLMs’ accuracy on CLA-QA based on physician-assigned score. Bars show the mean physician-assigned scores over 25 answers for each answer-generation model. Error bars represent standard deviations, and the mean ± SD values are displayed above each bar for reference.

To enable consistent and scalable evaluation of LLM-generated free-text responses, we first identified a high-quality set of reference answers. Expert physicians rated GPT-4’s responses highest in accuracy (mean score 4.77, *SD = *0.25); therefore, we selected these responses as the reference set for the reference-guided evaluation methods (see Figure 3 and Table S3). After designating GPT-4’s responses as the reference, we excluded them from subsequent evaluation of free-text responses using automated methods. Among the remaining answer-generation models evaluated, Phi-4 achieved the highest average physician-assigned score of 3.53 (*SD = *1.25), while DeepSeek-R1-8B received the lowest average score (2.64, *SD = *1.33). The Friedman test showed a significant difference in physician-assigned scores across the six evaluated models (*p* = .005).

### Effectiveness of automated evaluation methods

#### Reference-based evaluation of NLP metrics and LLM evaluators

We computed NLP sentence similarity metrics and conducted reference-guided scoring with LLM evaluators using reference answers. To assess their alignment with expert judgment, we calculated the Spearman correlation between the resulting automatic evaluation scores and physician-assigned scores.

Among four NLP sentence similarity metrics assessed, METEOR had the lowest correlation with physician-assigned scores (Spearman *ρ* = 0.240), while BERTScore had the strongest correlation (*ρ* = 0.406). Among LLM evaluators, GPT-4 achieved the highest correlation with physician-assigned scores (*ρ* = 0.758), followed closely by GPT-4o (*ρ* = 0.727). All four open-source LLM evaluators demonstrated moderate correlations with physician-assigned scores, ranging from 0.506 (DeepSeek-R1-14B) to 0.608 (Qwen3) (Table 1).

**Table 1.** Correlation of NLP sentence similarity metrics and reference-guided LLM-based evaluation with physician assessment

	Correlation with Physician-Assigned Score	
	Spearman *ρ*	p value
***NLP Sentence Similarity Metrics***		
**ROUGE-L**	0.325	< 0.001
**BLEU**	0.361	< 0.001
**METEOR**	0.240	0.003
**BERTScore**	0.406	< 0.001
***LLM evaluator using Reference-guided Scoring***		
**GPT-4 Evaluator**	**0.758**	< 0.001
**GPT-4o Evaluator**	0.727	< 0.001
**Gemma3-27B Evaluator**	0.552	< 0.001
**DeepSeek-R1-14B Evaluator**	0.506	< 0.001
**Qwen3-32B Evaluator**	0.608	< 0.001
**LLaMA3.3-70B Evaluator**	0.596	< 0.001

* The highest score is shown in bold.

To further examine how these evaluation metrics differentiate free-text answer quality across models, we compared the mean accuracy scores of responses generated by different models using both classic NLP sentence similarity metrics with LLM-based reference-guided evaluators (Table 2). Across the six answer-generation models, NLP sentence similarity metrics exhibited relatively narrow score ranges across models. All NLP metrics captured some performance differences, they demonstrated statistically significant variation in model scores based on the Friedman test (*p* < .001), indicating their ability to differentiate among the evaluated models, with at least one model performing significantly better than the others. However, the resulting performance rankings were not aligned with physician-assigned scores. For instance, the DeepSeek-R1-8B model received the lowest average score from expert reviewers yet was rated relatively highly by several NLP metrics.

**Table 2.** Performance of answer-generation models evaluated by NLP metrics and LLM-based reference-guided scoring.

Evaluation Methods	Answer Generation Models						Statistical Test
	Phi-4-14B	Mistral-7B	Qwen2-7B	Gemma2-7B	LLaMA3.2-3.2B	DeepSeek-R1-8B	Friedman *p*
**Physician-Assigned Score**	3.53 ± 1.25	3.43 ± 1.22	3.40 ± 1.00	2.95 ± 1.07	2.93 ± 1.08	2.64 ± 1.33	0.005
***NLP Sentence Similarity Scores***							
**ROUGE-L**	0.23 ± 0.05	0.22 ± 0.05	0.21 ± 0.02	0.14 ± 0.04	0.22 ± 0.04	0.17 ± 0.02	< 0.001
**BLEU**	0.05 ± 0.04	0.03 ± 0.02	0.04 ± 0.02	0.01 ± 0.01	0.04 ± 0.04	0.02 ± 0.02	< 0.001
**METEOR**	0.32 ± 0.07	0.21 ± 0.05	0.34 ± 0.05	0.13 ± 0.06	0.28 ± 0.07	0.25 ± 0.07	< 0.001
**BERTScore**	0.88 ± 0.02	0.87 ± 0.02	0.87 ± 0.01	0.84 ± 0.02	0.87 ± 0.01	0.86 ± 0.02	< 0.001
***LLM-based Evaluation using Reference-guided Scoring***							
**GPT-4 Evaluator**	3.68 ± 1.35	3.28 ± 1.31	3.08 ± 0.86	3.12 ± 1.13	2.60 ± 0.76	2.52 ± 1.12	< 0.001
**GPT-4o Evaluator**	3.80 ± 1.22	3.60 ± 1.15	3.52 ± 0.82	3.40 ± 1.19	3.00 ± 0.71	2.80 ± 1.22	< 0.001
**Gemma3-27B Evaluator**	3.92 ± 1.00	3.96 ± 0.73	4.00 ± 0.65	3.80 ± 1.08	3.76 ± 0.60	3.16 ± 1.14	0.001
**DeepSeek-R1-14B Evaluator**	3.68 ± 0.99	3.56 ± 0.82	3.60 ± 0.71	3.40 ± 0.87	3.52 ± 0.82	3.04 ± 0.98	0.053
**Qwen3-32B Evaluator**	3.56 ± 1.19	3.44 ± 1.00	3.52 ± 0.92	3.36 ± 1.04	3.24 ± 0.78	2.56 ± 1.19	< 0.001
**LLaMA3.3-70B Evaluator**	3.76 ± 1.20	3.88 ± 1.05	3.88 ± 0.83	3.68 ± 1.25	3.64 ± 0.99	2.96 ± 1.43	0.011

* Scores are reported as mean ± standard deviation.

**Figure 4.**
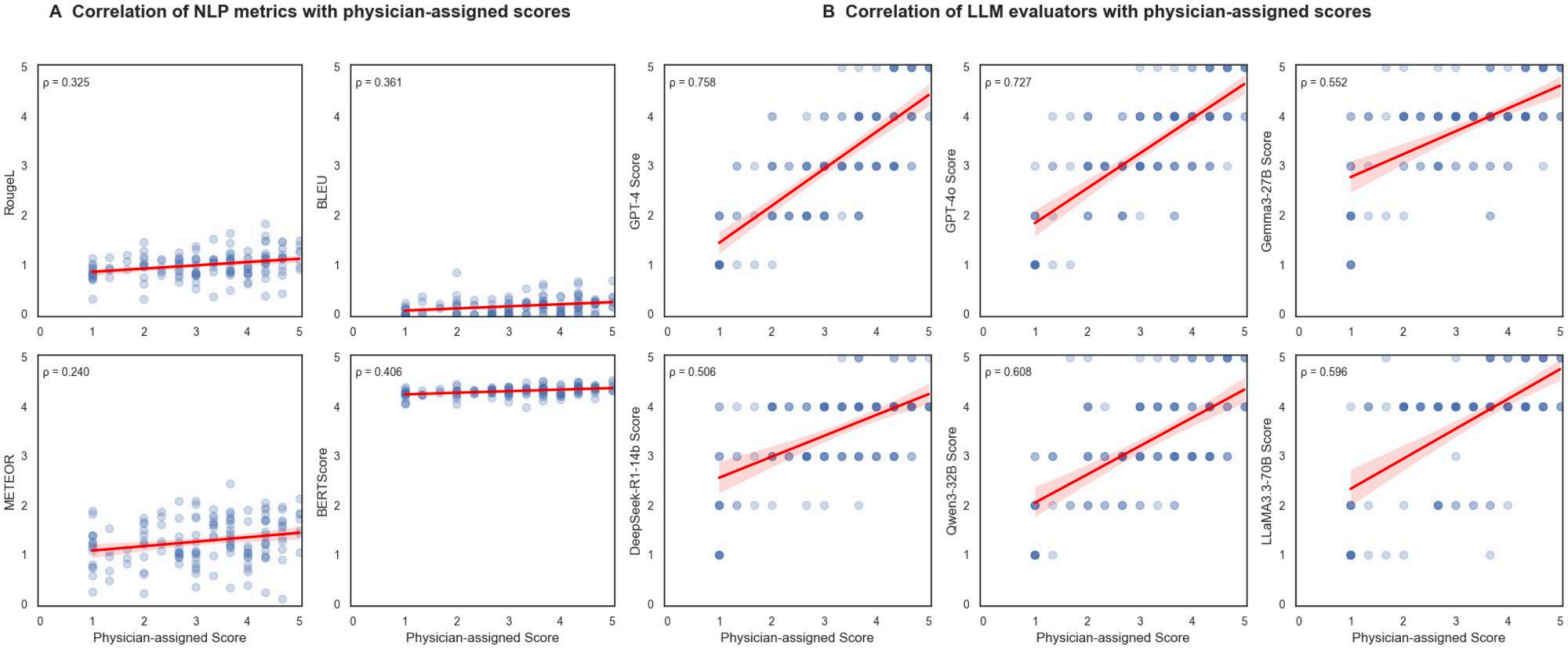
Scatter plots comparing automated evaluation scores with physician-assigned accuracy scores across all responses. The left panel displays correlations for traditional NLP metrics (BLEU, ROUGE-L, METEOR, and BERTScore), and the right panel presents correlations for LLM evaluators under the referenceguided setting. NLP scores were rescaled from the range 0–1 to 0–5 for consistency with the 5-point physician accuracy scale. Points with higher density appear darker due to repeated (or nearly repeated) values at that coordinate. Each subplot reports the Spearman correlation coefficient (*ρ*) in the upper-left corner. Darker points indicate higher density where multiple responses share similar scores. Red lines show linear regression fits to visualize overall alignment trends between automated metrics and expert ratings.

In comparison, all LLM evaluators exhibited variation in scoring answer-generation models. The GPT-4 evaluator assigned the highest average score of 3.68 (*SD = *1.35) to Phi-4, which was aligned closely with its highest physician-assigned average score of 3.53 (*SD = *1.25). Similarly, the GPT-4o evaluator assigned the highest score for Phi-4 (3.80, *SD = *1.22), followed by 3.60 (*SD = *1.15) for Mistral. Overall, Figure 4 illustrates that GPT evaluators closely align with physician-assigned scores, whereas NLP sentence similarity metrics diverge substantially, in terms of relative model ranking. Except for the DeepSeek-R1-14B evaluator (Friedman test, *p* = .053), all other LLM evaluators showed statistically significant differences in scores across answer-generation models (*p* < .05), indicating their ability to distinguish model performance.

#### Comparison across LLM-based evaluation methods

##### Reference-guided versus reference-free scoring

We next further characterized agreement between automated LLM evaluators and physician-assigned scores, both for reference-free scoring and reference-guided scoring.

When evaluating each response produced by the answer-generation models, GPT-4 and GPT-4o-assigned scores correlated more strongly with physician-assigned scores under reference-guided methods (*ρ* = 0.758 and 0.727, respectively) than under reference-free methods (*ρ* = 0.661 and 0.662, respectively). All other models also showed weaker correlations under reference-free compared to reference-guided methods, except for Qwen3, which demonstrated a slight increase in correlation with physician-assigned scores, from *ρ* = 0.608 to *ρ* = 0.614 (Table 3).

**Table 3.** Correlation of LLM-based evaluation methods with physician-assigned scores.

*Response Level (150 responses)*				
Evaluation Method	Reference-guided Scoring		Reference-free Scoring	
*LLM evaluators*	Spearman *ρ*^1^	**Binary Correlation** ^2^ *φ*	Spearman *ρ*	**Binary Correlation** *φ*
***GPT-4 Evaluator***	0.758	0.508	0.690	0.501
***GPT-4o Evaluator***	0.727	0.500	0.700	0.415
***Gemma3-27B Evaluator***	0.552	0.314	0.388	0.261
***DeepSeek-R1-14B Evaluator***	0.506	0.304	0.183	0.131
***Qwen3-32B Evaluator***	0.608	0.365	0.614	0.419
***LLaMA3.3-70B Evaluator***	0.596	0.349	0.409	0.179

*Answer Generation Model Level (6 models)*				
Evaluation Method	Reference-guided Scoring		Reference-free Scoring	
*LLM evaluators*	Kendall’s *τ*^1^	*p* Value	Kendall’s τ	*p* Value
***GPT-4 Evaluator***	0.867	0.020	0.867	0.020
***GPT-4o Evaluator***	1.000	0.003	0.867	0.017
***Gemma3-27B Evaluator***	0.600	0.136	0.690	0.136
***DeepSeek-R1-14B Evaluator***	0.733	0.056	0.690	0.136
***Qwen3-32B Evaluator***	0.867	0.017	0.966	0.017
***LLaMA3.3-70B Evaluator***	0.690	0.056	0.552	0.056

1 Binary Phi correlation was calculated by recoding the 5-point accuracy scale into two categories: low accuracy (scores 1–3) and high accuracy (scores 4–5).

2 All response-level correlation results were statistically significant (*p* < .05).

After recoding the 5-point scores into binary categories–low accuracy (1-3) and high accuracy (4-5)—both LLM-based reference-guided and reference-free methods showed reduced alignment with physician-assigned scores as measured by the Phi coefficient. Among the LLM evaluators, GPT-4o-assigned scores exhibited the strongest alignment with physician-assigned scores (*φ* = 0.508, moderate) under the reference-guided approach, whereas the DeepSeek-R1-14B evaluator showed the weakest correlation, indicating limited ability to distinguish between high- and low-quality responses in binary categorization.

**Figure 5.**
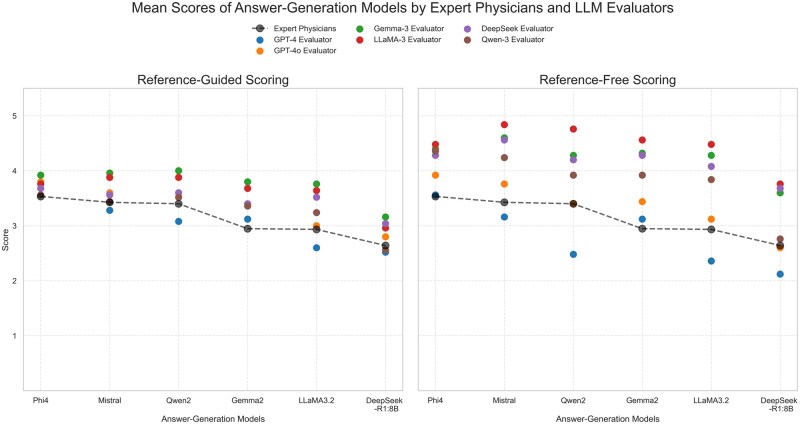
Comparison of reference-guided versus reference-free scores assigned by LLM evaluators across answer-generation models. Mean accuracy scores assigned to each answer-generation model by expert physicians and LLM evaluators using reference-guided (left) and reference-free (right) scoring methods. Scores are reported on a 5-point Likert scale. Each point represents the mean score assigned by either expert physicians, or an LLM evaluator for a given answer generation model, using the reference-guided scoring method. Black dashed lines represent physician-assigned scores; colored markers denote different LLM evaluators.

To assess the extent to which evaluators ranked each model’s overall performance similarly to physicians, we computed Kendall’s *τ* between the average physician-assigned scores and LLM-assigned scores at the model level. GPT-4 and GPT-4o evaluators demonstrated strong rank correlation with physicians’ judgments, with Kendall’s *τ* ranging from 0.867 to 1.000. Among open-source LLM evaluators, Qwen3 evaluator exhibited the strongest alignment with physician, achieving *τ* = 0.867 under reference-guided scoring and *τ* = 0.966 under reference-free scoring. Figure 5 illustrates the comparative results of both LLM-based evaluation methods across all LLM evaluators. Under the reference-free setting, LLM evaluators’ scores showed greater deviation from physician-assigned scores than under reference-guided scoring. Supplementary Table S4, available as supplementary data at [*JAMIA Open*] online reports the mean scores for each answer-generation model under the reference-free scoring setting.

### Evaluation design and robustness analysis in LLM-based evaluation

#### Stability of LLM-based evaluations

To assess the stability of LLM evaluators, we conducted 50 replicates of reference-guided scoring. The mean variance across all responses was 0.048 (*SD = *0.26) for the GPT-4o evaluator and 0.009 (*SD = *0.037) for the GPT-4 evaluator, indicating high overall stability. A notable exception was observed for a GPT-4-generated response regarding Kaposiform Lymphangiomatosis (KLA)’s hereditary risk that received a physician-assigned score of 5. For this response, the GPT-4o evaluator’s scores varied considerably across runs: 52% were rated as 5, 14% as 3, and 34% as 1. This highlights a potential limitation in scoring consistency when evaluating clinically complex answers. Meanwhile, all four open-source LLM evaluators demonstrated perfect scoring consistency (variance = 0) across 50 runs, due to deterministic outputs under a fixed temperature setting of zero.

#### Impact of prompt design

**Figure 6.**
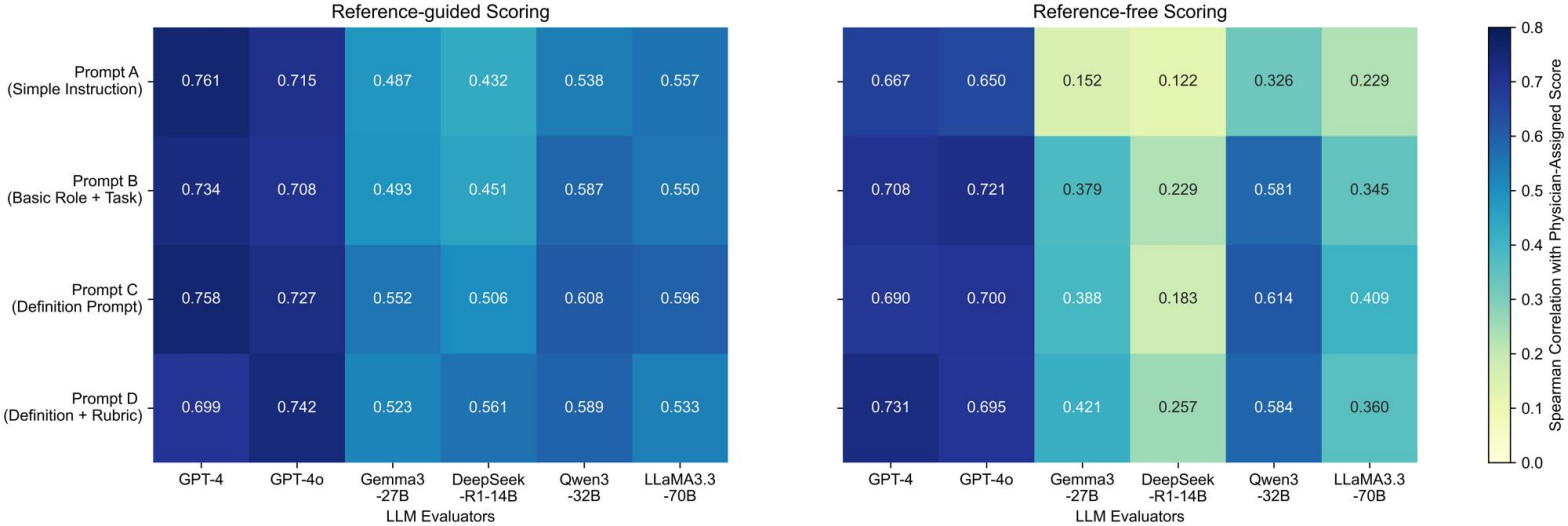
Effect of prompt variants on LLM evaluator performance measured by Spearman correlation with Physician-assigned scores. Heatmaps showing that GPT-4 and GPT-4o evaluators maintain consistently high correlations with physician-assigned scores across prompts, while open-source LLM evaluators vary more widely and show weaker correlations, especially in the reference-free setting.

To examine the sensitivity of LLM evaluators to prompt structure and complexity, we tested four prompt variants and assessed their impact on LLM-based evaluation performance using Spearman correlation coefficients (Figure 6). For GPT-4 and GPT-4o evaluators, prompt design had minimal influence on performance: correlations with physician-assigned scores remained consistently high across prompts (*ρ* = 0.699–0.761) under the reference-guided setting and moderately high under the reference-free setting (*ρ* = 0.667–0.731). By comparison, open-source LLM evaluators exhibited greater variability in correlation with physician-assigned scores depending on prompt design, particularly in the reference-free setting. Despite some improvement with more structured prompts, their correlations remained in the moderate or weak range.

#### Inference time and computation cost

To evaluate the feasibility of LLM-based evaluation, we recorded inference time and computation cost. GPT-4 and GPT-4o evaluators were the most time-efficient, averaging 1.68 (*SD = *0.66) and 1.51 (*SD = *0.57) seconds per responses, respectively. Open-source LLM evaluators such as Qwen3 and LLaMA3.3 required over 30 and 200 seconds per response (Supplementary Table S5, available as supplementary data at [*JAMIA Open*] online). Regarding computational cost, GPT-4 and GPT-4o evaluators incurred estimated per-response costs of (8.3 ± 1.4)×10^−^³ USD and (2.30 ± 0.37)×10^−^³ USD, respectively. Open-source LLM evaluators were run locally using Ollama and did not incur additional per-inference costs.

#### Bias in LLM-based evaluation

##### Verbosity bias

To investigate whether answer length influenced scores, we calculated the Spearman correlation between LLM-assigned scores and answer length. Correlations ranged from 0.025 (GPT-4) to 0.162 (DeepSeek-R1-14B), suggesting weak verbosity effects (Supplementary Table S6, available as supplementary data at [*JAMIA Open*] online). The correlation between physician-assigned scores and answer length was 0.167, indicating no significant association.

###### Self-enhanced bias

We examined whether LLM evaluators favored their own model family under reference-guided scoring (Table 2). The Gemma3 evaluator assigned higher scores to Gemma2 responses (3.80, *SD = *1.08), which exceeded the physician-assigned rating 2.95 (*SD = *1.07). DeepSeek-R1-14B rated its own model’s responses at 3.04(*SD = *0.98), compared to a lower physician-assigned score of 2.64 (*SD = *1.33). Similarly, the LLaMA3.3 evaluator gave a score of 3.64 (*SD = *0.87) to LLaMA3.2 responses, also higher than the physician score of 2.93(*SD = *1.08). Qwen3 evaluator did assigned a score of 3.52 to Qwen2 responses, close to the physician-assigned score of 3.40. However, while these LLM evaluators assigned higher scores to their own models compared to physician ratings, they also consistently gave elevated scores across all models, indicating general score inflation rather than targeted self-favoritism.

## Discussion

With the growing need to develop LLM-based chatbots for rare disease patients and families, we must address the challenge of assessing the quality of large volumes of free-text responses generated by these systems. Because human evaluation is not feasible at scale, automation becomes essential. Automating the evaluation of LLM-generated responses is a critical yet challenging task, given the clinical importance of ensuring nuanced and accurate information to meet the needs of rare disease patients and families. In this study, we systematically compared traditional NLP sentence similarity metrics with LLM-based evaluation methods to assess their effectiveness in approximating expert physicians’ judgment of the accuracy of LLM output. Our findings provide valuable insights into the performance, reliability, and limitations of automated evaluation methods.

Our results showed that LLM-based evaluation outperformed traditional NLP metrics in aligning with physician-assigned accuracy scores. Proprietary LLM evaluators, particularly GPT-4 and GPT-4o, demonstrated the strongest response-level correlations with physician ratings across both reference-guided and reference-free settings. In contrast, commonly used NLP metrics, including ROUGE-L, BLEU, METEOR and BERTScore, exhibited consistently weaker correlations with physician-assigned scores. Although these metrics are frequently applied in medical QA when reference answers are available, they fundamentally measure lexical or semantic similarity rather than factual correctness.^22^^,^^61^ Including them as comparators highlights the limitations of using similarity as a proxy for answer quality in clinical contexts. While these metrics detected statistically significant differences among answer-generation models, their resulting rankings did not consistently align with expert judgment, underscoring the need for evaluation frameworks explicitly designed to assess factual accuracy.

The observed limitations of traditional NLP metrics likely stem from their underlying assumptions. N-gram-based metrics emphasize surface-level overlap, while embedding-based metrics such as BERTScore rely on pretrained representations that may inadequately capture rare disease-specific concepts. These embedding models are typically fixed and not optimized for specialized medical domains, limiting their adaptability.^62^^,^^63^ Moreover, reference-based NLP metrics require high-quality gold-standard answers, which are difficult to produce at scale for rare diseases. In contrast, LLM-based evaluation methods, particularly those using structured rubrics, can be applied in both reference-guided and reference-free settings, providing greater flexibility and scalability while more directly assessing factual correctness.

Among LLM evaluators, reference-guided scoring consistently yielded stronger alignment with physician-assigned scores than reference-free scoring. However, reference-free evaluation remains appealing because it does not require curated reference answers. In our study, reference-free evaluation showed greater score variability and weaker correlations with physician-assigned scores, particularly for open-source models. Both reference-guided and reference-free approaches performed less reliably in binary classification, suggesting that multi-point scoring rubrics more effectively capture the nuanced gradations of factual accuracy in clinical responses.

Open-source LLM evaluators generally demonstrated weaker alignment with physician ratings compared to proprietary GPT-based models. This performance gap likely reflects that open-source models are trained on smaller or less medically diverse corpora and receive less extensive medical grounding, alignment, and safety tuning than systems such as GPT-4.^16^^,^^37^^,^^48^^,^^64^ Open-source models may struggle to detect subtle factual inaccuracies in specialized clinical contexts, leading to greater score variability. Importantly, the evaluated models vary in size, release date, and training characteristics; therefore, comparisons should be interpreted as specific to this model set rather than as general conclusions about relative model performance. These findings do not diminish their potential utility; rather, they highlight opportunities for improvement through domain-specific fine-tuning, instruction tuning for evaluation tasks, and retrieval-augmented evaluation that incorporates authoritative clinical sources. Future work should directly assess whether these strategies can narrow the gap between open-source and proprietary LLM evaluators.

Stability analyses support the reproducibility of LLM-based evaluation under a fixed temperature setting of zero. While GPT-4 and GPT-4o evaluators were relatively robust to prompt variation, open-source models showed greater sensitivity to prompt design, particularly in reference-free settings. Although the use of GPT-4-generated reference answers may contribute to the stability of proprietary evaluators in reference-guided evaluation, prompt sensitivity among open-source models persisted even without reference answers. This suggests that differences in robustness are not solely driven by reference selection, but may instead reflect underlying differences in instruction-following behavior, alignment, or domain knowledge. These findings underscore the need for further research to identify optimal prompt designs for evaluation tasks.

We observed minimal evidence of verbosity bias across evaluators, with weak correlations between response length and evaluation scores. However, this analysis was exploratory and did not isolate verbosity as an independent factor. Prior work has demonstrated that controlled manipulations of verbosity can reveal systematic biases in LLM evaluators.^28^ Applying similar controlled paradigms in future studies would allow for a more rigorous assessment of verbosity effects in patient-facing clinical QA. We also observed modest score inflation when evaluators assessed responses generated by their the same family, particularly among open-source systems. This pattern appeared to reflect general score inflation rather than targeted self-enhancement. Further research is needed to investigate these biases in more depth.

Qualitative analysis provided additional insight into the strengths and limitations of LLM-based evaluation. Using the GPT-4 reference-guided evaluator as an illustrative example (Supplementary Table S7, available as supplementary data at [*JAMIA Open*] online), we found that false positives, in which the evaluator assigned high accuracy scores to responses that were well structured but contained clinically important inaccuracies, such as incorrect statements about disease mechanisms or inheritance patterns. False negatives occurred when evaluators penalized cautious or imperfect phrasing more heavily than clinicians, despite the overall clinical message being acceptable. These cases illustrate that LLM evaluators capture broad trends in factual accuracy but may inconsistently weight nuanced clinical details, reinforcing the need for clearer rubrics, calibration, and domain-specific tuning.

Overall, these findings highlight the potential of LLM-based evaluation, particularly GPT-4 and GPT-4o models, to support large-scale, automated assessment of LLM-generated responses in rare disease contexts. High correlations with physician ratings and short inference times suggest that these evaluators could substantially reduce the burden of manual review while preserving clinically meaningful signal. Reference-guided LLM evaluation emerged as the most reliable approach in this study, though reference-free methods remain valuable when reference answers are unavailable.

Despite these promising results for LLM-based evaluation, several limitations must be acknowledged. First, our evaluation focused on 25 questions related exclusively to CLAs. While this domain provides a clinically coherent testbed, it limits generalizability to other rare diseases and medical domains. Future work will expand this framework to more diverse diseases and incorporate questions drawn directly from patient-facing platforms. Second, physician annotations though provided by board-certified experts, inherently involve subjective judgment. Aggregating ratings mitigates but does not eliminate variability.

Third, while we assessed rank correlation between automated and physician-assigned scores, we did not evaluate score calibration. High rank agreement does not guarantee that absolute score magnitudes align with clinical expectations. Future studies should incorporate calibration analyses and clinician-verified scoring anchors to improve interpretability. Fourth, this study focused exclusively on accuracy to establish a foundational evaluation framework. Other important dimensions of patient-facing responses, such as completeness, relevance, clarity, empathy and readability, were outside the scope of this analysis but warrant systematic evaluation in future work.

Finally, we used GPT-4-generated answers as reference answers for reference-guided evaluations. Although GPT-4 outputs are rated highly by physicians, they may introduce subtle model-specific biases. Importantly, strong alignment between LLM evaluators and physician ratings persisted in the reference-free setting, suggesting that evaluator performance was not solely driven by similarity to GPT-4 references. Nonetheless, reference-guided evaluation is not fully automated, as it requires selecting reliable reference answers. In real-world deployments, physician-written references, curated knowledge bases, or automatically selected high-quality answers could replace GPT-4 outputs. Automating reference selection represents an important direction for future work.

Future work may also explore hybrid evaluation approaches that combine LLM-based evaluation with traditional metrics or external knowledge verification to further improve reliability and robustness. Together, these findings provide a foundation for developing scalable, clinically meaningful evaluation frameworks for LLM-generated responses in rare disease and other patient-facing medical applications.

## Conclusion

To the best of our knowledge, this study is the first to demonstrate the application of automated LLM-based evaluation methods for free-text responses in the rare disease domain. Our results highlighted the potential of LLM-based evaluation, particularly GPT series models, as scalable, automated alternatives to human review in evaluating LLM-generated responses to patient questions about rare diseases. While LLM-based evaluation approaches show promising results, they must be carefully designed, validated, and continuously monitored to ensure their outputs remain clinically relevant and aligned with human judgment. Future work should further explore LLM-based evaluation robustness and generalizability across diverse rare disease domains.

## Supplementary Material

ooag054_Supplementary_Data

## Data Availability

The CLA-QA dataset, consisting of 25 common CLA patient questions, 175 responses generated by seven LLMs, and accuracy annotations from three expert physicians, is available in the Washington University institutional repository at https://data.library.wustl.edu/record/108301? v=tab.

## Code availability

A public repository containing the full prompt templates and example evaluation scripts used in this study is available at https://github.com/Min-Zhao/llm-eval-rare-disease.

## References

[1] Definition of rare disease—NCI Dictionary of Cancer Terms—NCI. 2011. Accessed June 16, 2025. https://www.cancer.gov/publications/dictionaries/cancer-terms/def/rare-disease

[2] Monaco L , Zanello G, Baynam G, et al Research on rare diseases: ten years of progress and challenges at IRDiRC. Nat Rev Drug Discov. 2022;21:319-320. 10.1038/d41573-022-00019-z 35079160 PMC7613920

[3] Zhu X , Smith RA, Parrott RL, et al Understanding information sharing about rare diseases: an evaluation of the NIH’s website on AATD. J Commun Healthc. 2018;11:128-139. 10.1080/17538068.2018.1453434

[4] Schieppati A , Henter J-I, Daina E, et al Why rare diseases are an important medical and social issue. Lancet. 2008;371:2039-2041. 10.1016/S0140-6736(08)60872-7 18555915

[5] Sisk BA , Kerr A, King KA. Factors affecting pathways to care for children and adolescents with complex vascular malformations: parental perspectives. Orphanet J Rare Dis. 2022;17:271. 10.1186/s13023-022-02432-4 35840977 PMC9287854

[6] Sisk B , Bereitschaft C, Kerr A. Communication with parents and young adult patients affected by complex vascular malformations. Pediatr Blood Cancer. 2023;70:e30158. 10.1002/pbc.30158 36545911

[7] Kalbfell R , Wang W, Fishman S, et al Burdens of disease and caregiver burden in complex vascular malformations. Pediatr Blood Cancer. 2023;70:e30367. 10.1002/pbc.30367 37114758

[8] Babac A , von Friedrichs V, Litzkendorf S, et al Integrating patient perspectives in medical decision-making: a qualitative interview study examining potentials within the rare disease information exchange process in practice. BMC Med Inform Decis Mak. 2019;19:188. 10.1186/s12911-019-0911-z 31533712 PMC6751820

[9] Udelnow A , Hecht V, Buschmann I, et al Disease knowledge and patient education are key players for a better quality of life in vascular surgery patients. Eur Surg. 2021;53:75-83. 10.1007/s10353-020-00684-7

[10] Adachi T , El-Hattab AW, Jain R, et al Enhancing equitable access to rare disease diagnosis and treatment around the world: a review of evidence, policies, and challenges. Int J Environ Res Public Health. 2023;20:4732. 10.3390/ijerph20064732 36981643 PMC10049067

[11] Mirpuri KK , Sisk B, Bereitschaft C, et al The role of perceived health‐related information adequacy in the experiences of parents of children with complex vascular anomalies. Pediatr Blood Cancer. 2025;72:e31697. 10.1002/pbc.31697 40172173

[12] Alkhulaifat D , Ramirez-Suarez KI, Otero HJ, et al Complex lymphatic anomalies. Pediatr Radiol. 2025;55:2348-2355. 10.1007/s00247-025-06167-9 39853393

[13] Kerr AM , Lin S, Sisk BA. Mental and physical health of adult patients affected by complex vascular anomalies. Patient Educ Couns. 2023;117:107987. 10.1016/j.pec.2023.107987 37769517

[14] Kerr AM , Bereitschaft C, Duty KM, et al Navigating care for rare diseases: Caregiver and patient advice for families and clinicians managing care for vascular malformations. Patient Educ Couns. 2023;107:107569. 10.1016/j.pec.2022.11.011 36410314

[15] Kerr AM , Wehrli J, Contente C, et al Dyadic coping experiences of parents of children with vascular anomalies. Pediatr Blood Cancer. 2024;71:e31261. 10.1002/pbc.31261 39171558

[16] Thirunavukarasu AJ , Ting DSJ, Elangovan K, et al Large language models in medicine. Nat Med. 2023;29:1930-1940. 10.1038/s41591-023-02448-8 37460753

[17] Arora RK , Wei J, Hicks RS, et al HealthBench: Evaluating Large Language Models Towards Improved Human Health [Internet]. arXiv; 2025 [cited 2026 Apr 6]. Available from: http://arxiv.org/abs/2505.08775; 10.48550/arXiv.2505.08775

[18] Kim JK , Chua M, Rickard M, et al ChatGPT and large language model (LLM) chatbots: the current state of acceptability and a proposal for guidelines on utilization in academic medicine. J Pediatr Urol. 2023;19:598-604. 10.1016/j.jpurol.2023.05.018 37328321

[19] Croxford E , Gao Y, First E, et alAutomating Evaluation of AI Text Generation in Healthcare with a Large Language Model (LLM)-as-a-Judge. medRxiv. 2025 May 6;2025.04.22.25326219. 10.1101/2025.04.22.25326219 PubMed PMID: 40313300; PubMed Central PMCID: PMC12045442.

[20] Zhang Y , Li Y, Cui L, et al Siren’s song in the AI Ocean: a survey on hallucination in large language models. [Internet]. arXiv; 2025 [cited 2026 Apr 6]. Available from: http://arxiv.org/abs/2309.01219; 10.48550/arXiv.2309.01219

[21] Gu J , Jiang X, Shi Z, et al A survey on LLM-as-a-judge. [Internet]. arXiv; 2025 [cited 2026 Apr 6]. Available from: http://arxiv.org/abs/2411.15594; 10.48550/arXiv.2411.15594

[22] Abbasian M , Khatibi E, Azimi I, et al Foundation metrics for evaluating effectiveness of healthcare conversations powered by generative AI. NPJ Digit Med. 2024;7:82-14. 10.1038/s41746-024-01074-z 38553625 PMC10980701

[23] Johri S , Jeong J, Tran BA, et al An evaluation framework for clinical use of large language models in patient interaction tasks. Nat Med. 2025;31:77-86. 10.1038/s41591-024-03328-5 39747685

[24] Lin C-Y. ROUGE: A Package for Automatic Evaluation of Summaries. Text Summarization Branches Out. Association for Computational Linguistics 2004:74-81.

[25] Papineni K , Roukos S, Ward T, et al BLEU: a method for automatic evaluation of machine translation. In: *Proceedings of the 40th Annual Meeting on Association for Computational Linguistics*. Association for Computational Linguistics 2002:311–318.

[26] Banerjee S , Lavie A. METEOR: An automatic metric for MT evaluation with improved correlation with human judgments. In: Goldstein J, Lavie A, Lin C-Y, et al, eds. Proceedings of the ACL Workshop on Intrinsic and Extrinsic Evaluation Measures for Machine Translation and/or Summarization. Association for Computational Linguistics; 2005:65-72.

[27] Zhang T , Kishore V, Wu F, et al BERTScore: Evaluating text generation with BERT.[Internet]. arXiv; 2020 [cited 2026 Apr 6]. Available from: http://arxiv.org/abs/1904.09675; 10.48550/arXiv.1904.09675

[28] Zheng L , Chiang W-L, Sheng Y, et al Judging LLM-as-a-judge with MT-bench and Chatbot Arena. In: Proceedings of the 37th International Conference on Neural Information Processing Systems. Red Hook, NY, USA: Curran Associates Inc.; 2023. p. 46595–623. (NIPS ’23).

[29] Chiang WL, Zheng L, Sheng Y, Angelopoulos AN, Li T, Li D, et al. Chatbot arena: an open platform for evaluating LLMs by human preference. In: Proceedings of the 41st International Conference on Machine Learning. Vienna, Austria: JMLR.org; 2024. p. 8359–88. (ICML’24).

[30] Zhang X , Yu B, Yu H, et al Wider and deeper LLM networks are Fairer LLM evaluators. [Internet]. arXiv; 2023 [cited 2026 Apr 6]. Available from: http://arxiv.org/abs/2308.01862; 10.48550/arXiv.2308.01862

[31] Adlakha V , BehnamGhader P, Lu XH, et al Evaluating Correctness and Faithfulness of Instruction-Following Models for Question Answering. Transactions of the Association for Computational Linguistics. 2024;12:681–99. 10.1162/tacl_a_00667

[32] Bavaresco A , Bernardi R, Bertolazzi L, et al LLMs instead of Human Judges? A Large Scale Empirical Study across 20 NLP Evaluation Tasks. In: Che W, Nabende J, Shutova E, Pilehvar MT, editors. Proceedings of the 63rd Annual Meeting of the Association for Computational Linguistics (Volume 2: Short Papers) [Internet]. Vienna, Austria: Association for Computational Linguistics; 2025 [cited 2026 Apr 6]. p. 238–55. Available from: https://aclanthology.org/2025.acl-short.20/ doi:10.18653/v1/2025.acl-short.20

[33] Gao M , Ruan J, Sun R, et al Human-like Summarization Evaluation with ChatGPT [Internet]. arXiv; 2023 [cited 2026 Apr 6]. Available from: http://arxiv.org/abs/2304.02554; 10.48550/arXiv.2304.02554

[34] Van Veen D , Van Uden C, Blankemeier L, et al Adapted large language models can outperform medical experts in clinical text summarization. Nat Med. 2024 Apr;30(4):1134–42. 10.1038/s41591-024-02855-5 PubMed PMID: 38413730; PubMed Central PMCID: PMC11479659. PMC11479659 38413730

[35] Weber MT , Noll R, Marchl A, et al MedBot vs RealDoc: efficacy of large language modeling in physician-patient communication for rare diseases. J Am Med Inform Assoc. 2025;32:775-783. 10.1093/jamia/ocaf034 39998911 PMC12012358

[36] OpenAI, Achiam J, Adler S, et al GPT-4 Technical Report [Internet]. arXiv; 2023 [cited 2026 Apr 6]. Available from: http://arxiv.org/abs/2303.08774; 10.48550/arXiv.2303.08774

[37] Nori H , King N, McKinney SM, et al Capabilities of GPT-4 on Medical Challenge Problems [Internet]. arXiv; 2023 [cited 2026 Apr 6]. Available from: http://arxiv.org/abs/2303.13375; 10.48550/arXiv.2303.13375

[38] Grattafiori A , Dubey A, Jauhri A, et al The Llama 3 Herd of Models [Internet]. arXiv; 2024 [cited 2026 Apr 6]. Available from: http://arxiv.org/abs/2407.21783; 10.48550/arXiv. 2407.21783

[39] DeepSeek AI , Guo D, Yang D, et al DeepSeek-R1: Incentivizing Reasoning Capability in LLMs via Reinforcement Learning. Nature. 2025 Sep 18;645(8081):633–8. 10.1038/s41586-025-09422-z PMC12443585 40962978

[40] Abdin M , Aneja J, Behl H, et al Phi-4 Technical Report [Internet]. arXiv; 2024 [cited 2026 Apr 6]. Available from: http://arxiv.org/abs/2412.08905; 10.48550/arXiv.2412.08905

[41] Jiang AQ , Sablayrolles A, Mensch A, et al. Mistral 7B [Internet]. arXiv; 2023 [cited 2026 Apr 6]. Available from: http://arxiv.org/abs/2310.06825; 10.48550/arXiv.2310.06825

[42] Team G , Riviere M, Pathak S, et al Gemma 2: Improving Open Language Models at a Practical Size. [Internet]. 2024 [cited 2026 Apr 6]. Available from: https://arxiv.org/abs/ 2408.00118v3; 10.48550/arXiv.2408.00118

[43] Yang A , Yang B, Hui B, et al Qwen2 Technical Report. [Internet]. 2024 [cited 2026 Apr 6]. Available from: https://arxiv.org/abs/2407.10671v4; 10.48550/arXiv.2407.10671

[44] Goodman RS , Patrinely JR, Stone CA Jr, et al Accuracy and reliability of chatbot responses to physician questions. JAMA Netw Open. 2023;6:e2336483. https://arxiv.org/abs/2403.16950v5; 10.48550/arXiv.2403.16950 37782499 PMC10546234

[45] Liu Y , Zhou H, Guo Z, et al Aligning with Human Judgement: The Role of Pairwise Preference in Large Language Model Evaluators. [Internet]. 2024 [cited 2026 Apr 6]. Available from: https://arxiv.org/abs/2403.16950v5 doi:10.48550/arXiv.2403.16950

[46] Liu Y , Iter D, Xu Y, et al G-Eval: NLG Evaluation using Gpt-4 with Better Human Alignment. Proceedings of the 2023 Conference on Empirical Methods in Natural Language Processing [Internet]. Singapore: Association for Computational Linguistics; 2023 [cited 2026 Apr 6]. p. 2511–22. Available from: https://aclanthology.org/2023.emnlp-main.153/; 10.18653/v1/2023.emnlp-main.153

[47] Sheng S , Xu Y, Fu L, et al Is Reference Necessary in the Evaluation of NLG Systems? When and Where? Proceedings of the 2024 Conference of the North American Chapter of the Association for Computational Linguistics: Human Language Technologies (Volume 1: Long Papers) [Internet]. Mexico City, Mexico: Association for Computational Linguistics; 2024 [cited 2026 Apr 6]. p. 8580–96. Available from: http://arxiv.org/abs/2410.21276; 10.18653/v1/2024.naacl-long.474

[48] OpenAI, Hurst A, Lerer A, et al GPT-4o System Card [Internet]. arXiv; 2024 [cited 2026 Apr 6]. Available from: http://arxiv.org/abs/2410.21276; 10.48550/arXiv.2410.21276

[49] Team G , Kamath A, Ferret J, et al Gemma 3 Technical Report [Internet]. arXiv; 2025 [cited 2026 Apr 6]. Available from:http://arxiv.org/abs/2503.19786; 10.48550/arXiv.2503.19786

[50] Yang A , Li A, Yang B, et al Qwen3 Technical Report [Internet]. arXiv; 2025 [cited 2026 Apr 6]. Available from: http://arxiv.org/abs/2505.09388; 10.48550/arXiv.2505.09388

[51] Ollama. https://ollama.com (accessed 4 April 2025)

[52] Saito K , Wachi A, Wataoka K, et al Verbosity Bias in Preference Labeling by Large Language Models [Internet]. arXiv; 2023 [cited 2026 Apr 6]. Available from: http://arxiv.org/abs/2310.10076; 10.48550/arXiv.2310.10076

[53] Fisher RA. Statistical methods for research workers. In: Kotz S, Johnson NL, eds. Breakthroughs in Statistics: Methodology and Distribution. Springer; 1992:66-70.

[54] Koo TK , Li MY. A guideline of selecting and reporting intraclass correlation coefficients for reliability research. J Chiropr Med. 2016;15:155-163. 10.1016/j.jcm.2016.02.012 27330520 PMC4913118

[55] Spearman C. The proof and measurement of association between two things. Am J Psychol. 1904;15:72-101. 10.2307/1412159 3322052

[56] Schober P , Boer C, Schwarte LA. Correlation coefficients: Appropriate use and interpretation. Anesth Analg. 2018;126:1763-1768. 10.1213/ANE.0000000000002864 29481436

[57] Kendall MG. A new measure of rank correlation. Biometrika. 1938;30:81-93. 10.1093/biomet/30.1-2.81

[58] Daniel WW. Friedman two-way analysis of variance by ranks. In Applied Nonparametric Statistics. PWS-Kent; 1990:262-74.

[59] Matthews BW. Comparison of the predicted and observed secondary structure of T4 phage lysozyme. Biochim Biophys Acta. 1975;405:442-451. 10.1016/0005-2795(75)90109-9 1180967

[60] Hogan RJ , Mason IB. Deterministic forecasts of binary events. In: Forecast Verification. John Wiley & Sons, Ltd; 2011:31–59.

[61] Shor J , Bi RA, Venugopalan S, et al Clinical BERTScore: an improved measure of automatic speech recognition performance in clinical settings. In: Naumann T, Ben Abacha A, Bethard S, et al, eds. Proceedings of the 5th Clinical Natural Language Processing Workshop. Association for Computational Linguistics; 2023:1-7.

[62] Michalopoulos G , Wang Y, Kaka H, et al UmlsBERT: Clinical Domain Knowledge Augmentation of Contextual Embeddings Using the Unified Medical Language System Metathesaurus. Proceedings of the 2021 Conference of the North American Chapter of the Association for Computational Linguistics: Human Language Technologies [Internet]. Online: Association for Computational Linguistics; 2021 [cited 2026 Apr 6]. p. 1744–53. Available from: https://aclanthology.org/2021.naacl-main.139/; 10.18653/v1/ 2021.naacl-main.139

[63] Reimers N , Gurevych I. Sentence-BERT: Sentence Embeddings using Siamese BERT-Networks. Proceedings of the 2019 Conference on Empirical Methods in Natural Language Processing and the 9th International Joint Conference on Natural Language Processing (EMNLP-IJCNLP) [Internet]. Hong Kong, China: Association for Computational Linguistics; 2019 [cited 2026 Apr 6]. p. 3982–92. Available from: https://aclanthology.org/D19-1410/; 10.18653/v1/D19-1410

[64] Singhal K , Azizi S, Tu T, et al Large language models encode clinical knowledge. Nature. 2023;620:172-180. 10.1038/s41586-023-06291-2 37438534 PMC10396962

[65] Devlin J , Chang M-W, Lee K, et al BERT: Pre-training of Deep Bidirectional Transformers for Language Understanding. Proceedings of the 2019 Conference of the North American Chapter of the Association for Computational Linguistics: Human Language Technologies, Volume 1 (Long and Short Papers) [Internet]. Minneapolis, Minnesota: Association for Computational Linguistics; 2019 [cited 2026 Apr 6]. p. 4171–86. Available from: https://aclanthology.org/N19-1423; 10.18653/v1/N19-1423

