# Supplementary material for: Automating evaluation of LLM-generated responses to patient questions about rare diseases
Source: JAMIA Open. 2026 Apr 17;9(2):ooag054. doi: 10.1093/jamiaopen/ooag054 (PMC13089572; doi:10.1093/jamiaopen/ooag054)
Supplement: ooag054_Supplementary_Data [file ooag054_supplementary_data.docx]

## SUPPLEMENTARY MATERIALS

### Table S1. CLA-QA dataset question list

| **CLA Questions** |
| --- |
| 1. What is Gorham Stout Disease? |
| 1. Is Gorham Stout Disease hereditary? |
| 1. What can make Gorham Stout Disease become active? Trauma, infection, puberty, pregnancy,… |
| 1. Why is having surgery or a biopsy risky for a Gorham-Stout Disease patient? Why can they not remove the lymphatic mass? |
| 1. Does generalized lymphatic anomaly seem to stay localized to the parts of the body that were originally affected or does it spread? |
| 1. What are the reported side effects of sirolimus for generalized lymphatic anomaly? |
| 1. Is it dangerous to have children if I have kaposiform lymphangiomatosis? |
| 1. Why do they have to use drug combinations for a kaposiform lymphangiomatosis patient? |
| 1. Does Lymphatic Drainage Massage work for central conducting lymphatic anomaly? |
| 1. Low fat diets - do they work for central conducting lymphatic anomaly? |
| 1. What do lymphatics do in your body? |
| 1. What type of imaging do I need for Gorham Stout Disease? How often do I need to get imaging? |
| 1. Does Lymphatic Drainage Massage work for Gorham-Stout Disease? |
| 1. How long can/should a Gorham-Stout Disease patient be on sirolimus? Can it be used for life? Should I/my child be monitored whilst on this drug? What does that look like? |
| 1. How is generalized lymphatic anomaly diagnosed? |
| 1. What genes cause generalized lymphatic anomaly? Does generalized lymphatic anomaly run in families? |
| 1. What are the symptoms of generalized lymphatic anomaly? |
| 1. What types of doctors take care of patients with Kaposiform lymphangiomatosis? |
| 1. What type of imaging do I need for kaposiform lymphangiomatosis? How often do I need to get imaging? |
| 1. What are the symptoms of kaposiform lymphangiomatosis? |
| 1. Why do we get so much pain with kaposifor lymphangiomatosis? |
| 1. Vaccine - Is it safe for me/my child to get the COVID vaccine given our diagnosis of kaposiform lymphangiomatosis? Should we receive the flu and pneumonia vaccine? Can we have live vaccines? Could a vaccine trigger the disease to become active? |
| 1. What are the symptoms of central conducting lymphatic anomaly? |
| 1. How long can/should a central conducting lymphatic anomaly patient be on sirolimus? Can it be used for life? Should I/my child be monitored whilst on this drug? What does that look like? |
| 1. Should I be taking supplements if I have central conducting lymphatic anomaly? If so, what? |

### Table S3. Example of rater disagreement for a response

Question: “What can make Gorham Stout Disease become active? Trauma, infection, puberty, pregnancy…”

Answer-generation model: Mistral-7B

Response: “Gorham-Stout syndrome (GSS), also known as vanishing bone disease or giant cell bone lesion, is a rare and progressive disorder characterized by the dissolution of bones, mostly in long bones and the pelvis. The exact cause of GSS is unknown, but it's often associated with the overproduction of fibrovascular tissue caused by an abnormal lymphatic system.

Regarding your question, while trauma or infection can cause temporary bone lesions, they are not known to trigger Gorham-Stout disease specifically. Puberty and pregnancy may not have a direct effect on the activity of GSS either. However, it's important to note that these factors might contribute to the onset of symptoms in some cases due to hormonal changes or increased stress on affected bones. Always consult with a healthcare professional for accurate information regarding specific conditions.”

| **Physician** | **Accuracy Score (1-5)** | **Rationale** |
| --- | --- | --- |
| Physician A | 3 | Not also known as "giant cell bone lesion". Downplays potential triggers. |
| Physician B | 4 | This one registers uncertainty in the answer, and doesn't say anything specifically incorrect, but it would be more appropriate to say that these conditions likely do cause increased activity of disease. |
| Physician C | 2 | GSD is rare and can be progressive. The cause is unknown. |

Summary: Experts differed in how they interpreted factual inaccuracies versus incomplete or misleading framing, resulting in accuracy scores of 2, 3, and 4 for the same response.

### Section S1. Description of NLP sentence similarity metrics

*ROUGE* ***(Recall-Oriented Understudy for Gisting Evaluation)***:[24] ROUGE measures the overlap of n-grams, word sequences, and word pairs between generated and reference answers. In this study, we used ROUGE-L, a variant that calculates similarity based on the longest common subsequence and accounts for sentence-level structural similarity.

*BLEU* ***(Bilingual Evaluation Understudy)***:**[25]** Originally developed for machine translation, BLEU calculates the precision of n-grams (typically up to 4-grams) in the generated answers that also appear in the reference answer. It incorporates a brevity penalty to discourage overly short outputs, thereby favoring concise and accurate responses.

*METEOR* ***(Metric for Evaluation of Translation with Explicit ORdering)***:**[26]** METEOR improves on BLEU by incorporating both precision and recall of unigrams and by aligning words using stemming, synonyms, and paraphrase matching.

*BERTScore*:[27] A semantic similarity metric that uses contextual embeddings from pretrained BERT models to compare token-level similarity between generated and reference responses.[67] Unlike surface-level overlap metrics, BERTScore can capture deeper semantic meaning and paraphrasing.

### Section S2: Statistical Measures Used in This Study

**Intraclass Correlation Coefficient (ICC):** ICC quantifies the degree of agreement among multiple raters evaluating the same items. We used a two-way random-effects model (ICC2) to assess consistency across three physicians scoring each response.[54]

**Spearman’s ρ (rho):** Spearman’s **ρ** correlation assesses the monotonic relationship between two ordinal or continuous variables based on ranked data. It is robust to nonlinearity and non-normality. [55]

**Kendall’s τ (Tau):** Kendall’s τ is a nonparametric measure of rank correlation that quantifies the similarity between two ranking orders. It is less sensitive to tied ranks and non-normal distributions than Pearson correlation.[57]

**Phi (φ) Coefficient:** The φ coefficient measures association between two binary variables (e.g., “contains incorrect fact: yes/no”). It is equivalent to Pearson correlation for 2×2 contingency tables. [60]

**Friedman Test:** The Friedman test is a non-parametric statistical method used to detect differences in outcomes across three or more related groups. It is appropriate when the same set of items (e.g., LLM-generated responses) is evaluated repeatedly under different conditions (e.g., across multiple answer-generation models), and when assumptions of normality may not hold. The test assesses whether the median ranks differ significantly across the groups.[58]

### Table S3. Average accuracy scores assigned by expert physician reviewers.

| **Answer Generation Model** | **Reviewer-BAS** | **Reviewer-KH** | **Reviewer-SCC** | **Physician-Assigned Score (Mean)** |
| --- | --- | --- | --- | --- |
| **GPT-4** | 4.64±0.57 | 4.72±0.54 | 4.96±0.2 | 4.77±0.25 |
| **Phi-4-14B** | 3.20±1.41 | 3.76±1.30 | 3.64±1.58 | 3.53±1.25 |
| **Mistral-7B** | 3.00±1.41 | 3.60±1.22 | 3.68±1.44 | 3.43±1.22 |
| **Qwen2-7B** | 2.92±1.32 | 3.68±0.99 | 3.60±1.44 | 3.40±1.00 |
| **Gemma2-7B** | 2.48±1.29 | 3.44±1.19 | 2.92±1.32 | 2.95±1.07 |
| **LLaMA3.2-3.2B** | 2.48±1.36 | 3.24±1.13 | 3.08±1.26 | 2.93±1.08 |
| **DeepSeek-R1-8B** | 2.24±1.20 | 2.80±1.41 | 2.88±1.67 | 2.64±1.33 |
| **Overall** | **2.99±1.44** | **3.61±1.25** | **3.54±1.48** | **3.38±1.25** |

Table S4 summarizes the mean and standard deviation of accuracy scores assigned independently by three expert physician reviewers across all answer generation models. Scores reflect each reviewer’s assessment of the factual accuracy of LLM-generated answers on a 5-point Likert scale (1 = completely inaccurate; 5 = completely accurate). Physician-assigned score refers to the mean of the three expert physician reviewers' ratings per response.

### Table S4. Mean and standard deviation of scores assigned by LLM-based evaluators under the reference-free scoring setting for each answer-generation model

| **Evaluation Methods** | **Answer Generation Models** | | | | | | **Statistical Test** |
| --- | --- | --- | --- | --- | --- | --- | --- |
|  | **Phi-4-14B** | **Mistral-7B** | **Qwen2-7B** | **Gemma2-7B** | **Llama3.2-3.2B** | **DeepSeek-R1-8B** | **Friedman p** |
| **Physician-Assigned Score** | 3.53±1.25 | 3.43±1.22 | 3.40±1.00 | 2.95±1.07 | 2.93±1.08 | 2.64±1.33 | 0.005 |
| **GPT-4 Evaluator** | 3.56±1.36 | 3.16±1.46 | 2.48±0.87 | 3.12±1.27 | 2.36±0.76 | 2.12±0.93 | p < 0.001 |
| **GPT-4o Evaluator** | 3.92±1.29 | 3.76±1.16 | 3.40±0.96 | 3.44±1.19 | 3.12±1.01 | 2.60±1.15 | p < 0.001 |
| **Gemma3-27B Evaluator** | 4.40±1.04 | 4.60±0.58 | 4.28±0.84 | 4.32±0.95 | 4.28±0.68 | 3.60±1.32 | 0.015 |
| **DeepSeek-R1-14B Evaluator** | 4.28±0.68 | 4.56±0.58 | 4.20±0.58 | 4.28±0.68 | 4.08±0.70 | 3.68±1.03 | 0.007 |
| **Qwen3-32B Evaluator** | 4.36±1.15 | 4.24±0.88 | 3.92±1.08 | 3.92±1.15 | 3.84±0.90 | 2.76±1.33 | p < 0.001 |
| **Llama3.3-70B Evaluator** | 4.48±1.12 | 4.84±0.37 | 4.76±0.44 | 4.56±0.87 | 4.48±0.51 | 3.76±1.39 | 0.002 |

### Table S5. Inference time and cost per response for LLM evaluators (Mean ± SD)

| **LLM evaluators** | **Inference Time (s/response)** | **Cost* (USD/response)** |
| --- | --- | --- |
| **GPT-4 Evaluator** | 1.68 ± 0.66 | (8.30 ± 1.40) × 10⁻³ |
| **GPT-4o Evaluator** | 1.51 ± 0.57 | (2.30 ± 0.37) × 10⁻³ |
| **Gemma3-27B Evaluator** | 13.08±4.95 | - |
| **DeepSeek-R1-14B Evaluator** | 21.59±3.90 | - |
| **Qwen3-32B Evaluator** | 30.60±8.96 | - |
| **LLaMA3.3-70B Evaluator** | 203.43±19.06 | - |

* Cost is approximated based on the number of tokens used per response.

### Table S6. Verbosity bias in LLM-based evaluation: Spearman correlation between evaluator scores and answer length under reference-guided scoring.

| **Correlation** | **Answer Length (words)** | |
| --- | --- | --- |
|  | Spearman ρ | p value |
| **Physician-Assigned Score** | 0.131 | 0.1 |
| **GPT-4 Evaluator** | 0.025 | 0.8 |
| **GPT-4o Evaluator** | 0.044 | 0.6 |
| **Gemma3-27B Evaluator** | 0.008 | 0.9 |
| **DeepSeek-R1-14B Evaluator** | 0.162 | 0.05 |
| **Qwen3-32B Evaluator** | 0.079 | 0.3 |
| **LLaMA3.3-70B Evaluator** | 0.054 | 0.5 |

As shown in Table S7, the low correlation between evaluator scores and answer length suggests that verbosity bias is not a major concern.

### Table S7. Examples of Agreement and Disagreement Between LLM Evaluator and Physicians

*This table presents representative cases where the GPT-4 reference-guided evaluator either aligned with or diverged from physician-assigned accuracy scores, including both false-positive (overestimation) and false-negative (underestimation) patterns.* *Displayed responses are excerpts of the original model outputs, selected to highlight key features relevant to evaluation; full responses are available in the data repository referenced in the Data Availability section.*

| **Divergence type** | **Question** | **Answer-generation model** | **Generated answers excerpts** | **Mean physician-assigned score** | **GPT-4 reference-guided evaluator** | **Brief qualitative explanation** |
| --- | --- | --- | --- | --- | --- | --- |
| Concordant | Why is having surgery or a biopsy risky for a Gorham-Stout Disease patient? Why can they not remove the lymphatic mass? | GPT-4 | "Gorham-Stout Disease (GSD), also known as vanishing bone disease or phantom bone disease, is a rare condition characterized by the progressive loss of bony matrix and replacement with fibrous tissue, often accompanied by proliferation of lymphatic vessels.  Because GSD involves abnormal lymphatic proliferation, surgical intervention can lead to the opening of these fragile lymphatic channels. This can result in lymph leakage into surrounding areas, which can be difficult to manage..." | 5 | 5 | The evaluator’s score aligned fully with physician ratings because the answer accurately described the surgical risks in Gorham–Stout Disease, including lymphatic leakage, infection, disease progression, incomplete removal, and poor wound healing. The response contained no misinformation or speculative claims and closely matched established clinical understanding, leading both physicians and the evaluator to judge it as fully accurate. |
| Concordant | What do lymphatics do in your body? | Gemma 2-7B | "**Lymphatics do the following in your body:** … * **Return blood to the heart:** They transport deoxygenated blood from tissues and organs back to the heart." | 3 | 3 | The evaluator’s score matched physician ratings because both identified the same key issue: although the answer correctly described several core lymphatic functions (fluid balance, immune transport, fat absorption), it included notable false claim that lymphatics return deoxygenated blood to the heart. Both physicians and the evaluator recognized this as a substantive factual error, leading to a moderate accuracy score that reflected a mix of correct concepts and critical mistakes. |
| Concordant | What is Gorham Stout Disease? | DeepSeek-R1-8B | "The term "Gorham Stout" appears to be a conceptual construct that can be interpreted across various contexts, including medical, linguistic, and cultural dimensions. Without specific information about its origin or field of use, several potential interpretations arise: 1. **Medical Context**: It could refer to a specific disease or condition named after individuals or entities involved in its discovery or study..." | 1 | 1 | The evaluator correctly assigned the lowest score, matching physician ratings. The answer provides no factual content about Gorham–Stout Disease and instead delivers unrelated conceptual speculation. Since both the evaluator and physicians judged the response as wholly inaccurate, this case represents strong evaluator–expert concordance. |
| False positive | Does Lymphatic Drainage Massage work for Gorham-Stout Disease? | DeepSeek-R1-8B | "Based on the analysis, Lymphatic Drainage Massage (LDM) is not considered an effective treatment for Gorham-Stout Disease. Here's the breakdown: 1. **Understanding Gorham-Stout Disease**: It is a rare condition causing bone marrow failure and structural issues in bones, leading to complications like pain and deformation. 2. **What is LDM?**: A massage technique aimed at stimulating the lymphatic system to aid in waste removal and reduce edema (swelling). ..." | 2 | 4 | The evaluator overestimated accuracy by emphasizing the correct conclusion that lymphatic drainage massage is not an established treatment while missing a key factual error. The answer incorrectly states that Gorham–Stout Disease causes bone marrow failure, which physicians identified as inaccurate. The evaluator appeared influenced by the response’s coherent structure, resulting in an inflated score. |
| False positive | What type of imaging do I need for kaposiform lymphangiomatosis? How often do I need to get imaging? | Gemma 2-7B | "* **Ultrasound:** Initial evaluation to assess the size and location of tumors. * **Magnetic resonance imaging (MRI):** Detailed assessment of tumor extension,  * **Computed tomography (CT) scan:** ...  **Imaging frequency:** * **Baseline imaging:** Prior to treatment initiation. * **Follow-up imaging:**  * 6-12 weeks after treatment initiation to assess response..." | 2 | 4 | The evaluator focused on the generally correct imaging modalities mentioned in the answer while missing key clinical inaccuracies. The response incorrectly frames Kaposiform Lymphangiomatosis as a tumor-based condition and provides imaging intervals that are not evidence based for KLA. Physicians identified these errors as substantial, yet the evaluator gave a high score, likely influenced by the detailed structure and overall clinical tone of the answer. |
| False positive | Is Gorham Stout Disease hereditary? | LLaMA3.2-3.2B | "Gorham-Stout disease is a rare bone disorder characterized by the progressive replacement of bone tissue with lymphatic vessels and fat cells. While there is no clear evidence that Gorham-Stout disease is hereditary, it often appears in families where there are multiple affected individuals..." | 2 | 3 | The evaluator focused on the correct description of Gorham–Stout disease as a rare bone disorder but overlooked a key factual error. The answer incorrectly states that the disease often appears in families with multiple affected individuals, which contradicts clinical evidence that GSD is almost always sporadic. The evaluator appeared influenced by the otherwise coherent explanation, resulting in a score that was higher than physician ratings. |
| Fase negative | Why do they have to use drug combinations for a kaposiform lymphangiomatosis patient? | Mistral-7B | "Kaposiform Lymphangiomatosis (K-HL) is a rare and complex vascular tumor of infancy and childhood. It arises from the lymphatic system and its pathogenesis involves abnormalities in several signaling pathways, such as VEGF, PDGF, and TIE2.  The use of drug combinations for K-HL patients is primarily aimed at targeting multiple aspects of the tumor's growth and behavior simultaneously, as well as addressing any potential resistance mechanisms…" | 3.67 (3,4,4) | 2 | The evaluator assigned a low score because it heavily penalized the answer’s conflation of Kaposiform Lymphangiomatosis with a vascular tumor and its speculative drug combinations, which are not standard for KLA. Physicians, however, judged the response as moderately accurate overall, noting that while specific regimens and terminology were imperfect, the general concept that combination therapy is used to target multiple pathways was reasonable. As a result, the evaluator’s score was lower than expert ratings. |
| Fase negative | Why do we get so much pain with kaposiform lymphangiomatosis? | LLaMA3.2-3.2B | "Kaposiform lymphangiomatosis (KL) is a rare and serious condition that can cause significant pain, among other symptoms. 1. **Abnormal vascular formation**: In KL, there is an abnormal growth of blood vessels, which can lead to inflammation and tissue damage. 2. **Inflammation and edema**: The inflammation and swelling… can lead to increased sensitivity… 3. **Nerve damage**: KL can cause nerve damage… 4. **Spinal cord compression**: In some cases, the abnormal vascular formation can compress the spinal cord…" | 4.33 (4,4,5) | 3 | The evaluator underscored this response by focusing on speculative elements, such as spinal cord compression and systemic inflammation, which are not typical features of Kaposiform Lymphangiomatosis. Physicians, however, viewed the answer as largely accurate because it correctly described several mechanisms of KL-related pain, including inflammation, edema, and nerve involvement. The evaluator placed greater weight on omissions and overgeneralizations, leading to a lower score than expert ratings. |
| Fase negative | Why is having surgery or a biopsy risky for a Gorham-Stout Disease patient? Why can they not remove the lymphatic mass? | DeepSeek-R1-8B | "Gorham-Stout syndrome is a rare genetic disorder characterized by the breakdown of bone structure into fibrous tissue, leading to spontaneous fractures and severe symptoms.  1. **Fibrotic Tissue Characteristics**: The aggressive growth of fibrous tissue in Gorham-Stout makes it difficult to remove during surgery… 3. **Structural Instability**: The breakdown of bones and replacement with fibrous tissue weakens surrounding tissues, increasing the risk of complications during surgery…" | 3 | 2 | The evaluator underscored this answer by focusing on speculative language about genetics, fibrotic tissue, and treatment details while physicians viewed the response as partially acceptable. Although the answer contains inaccuracies and overextended claims, experts judged it as moderately accurate overall, whereas the evaluator treated these issues as more severe, resulting in a lower score than physician ratings. |

### Table S8. LLM evaluators model information

| **Model** | **Parameter Size** | | **Open-Source or Proprietary** | | **Release Date** | | **Model Architecture** | |
| --- | --- | --- | --- | --- | --- | --- | --- | --- |
| **GPT-4o Evaluator** | Not publicly disclosed | Proprietary | | 2024-05-13 | | Multimodal transformer (Omni) | |  |
| **GPT-4 Evaluator** | Not publicly disclosed | Proprietary | | 2023-03-14 | | Undisclosed | |  |
| **Gemma 3-27B Evaluator** | 27B | Open-Weights | | 2025-03-12 | | Decoder-only Transformer | |  |
| **DeepSeek-R1-14B Evaluator** | 14B | Open-Weights | | 2025-01-20 | | Dense Decoder-only Transformer | |  |
| **Qwen 3-32B Evaluator** | 32B | Open-Weights | | 2025-04-28 | | Dense Decoder-only Transformer | |  |
| **LLaMA 3.3-70B Evaluator** | 70B | Open-Weights | | 2024-12-06 | | Dense Decoder-only Transformer | |  |
